# Infraspinatus/Teres Minor Transfer Biceps In Situ Tenodesis Procedure: Initial Results of a Technique for Massive Cuff Tears

**DOI:** 10.1155/2013/646598

**Published:** 2013-10-30

**Authors:** Matt D. A. Fletcher

**Affiliations:** Department of Orthopaedics, Dawson Creek & District Hospital, 11100-13th Street, Dawson Creek, BC, Canada V1G 3W8

## Abstract

Massive rotator cuff tears may not be primarily repairable with salvage options not necessarily providing acceptable results. Extrinsic tendon transfer is a significant undertaking with prolonged rehabilitation and variable outcome. A novel technique for the reconstruction of massive tears, not amenable to primary repair, by performing a transfer of the intrinsic posterior rotator cuff onto an intact, tenodesed long head of biceps tendon acting as a scaffold for the intrinsic transfer is described. The clinical results at short to medium term in 17 initial patients are presented. Encouraging results from this study suggest that this is a viable option for the management of massive rotator cuff tears with an intact posterior cuff with results equal or superior to other reconstructive techniques.

## 1. Introduction

Rotator cuff pathology is a common problem, with tears causing a significant amount of time off work, pain, and loss of function. Tears can be graded according to a number of different systems, massive tears generally being considered the most difficult for surgical management. In the case of a massive tear with a retracted, deficient, or grossly degenerate tendon margin, direct primary repair is not possible. Present reconstructive options include medialisation of the supraspinatus footprint, margin convergence, subscapularis transfer, and extrinsic latissimus dorsi/teres major transfer.

A novel technique for the primary reconstruction of a massive, retracted, and irreparable tear is described. This utilizes the infraspinatus and teres minor muscles, as well as an intact long head of biceps to create a new, postero-superior force couple with a more anatomic force transmission alignment of the transferred tendon compared to an extrinsic transfer. The transferred tendon acts as a humeral head depressor as well as force couple to facilitate return to normal shoulder kinematics.

The procedure has the advantage of being performed as a primary operation and is easily accomplished in the deck-chair position. Recovery appears similar to simple rotator cuff repair, and postoperative pain is minimal. 

## 2. Methods

In deck-chair position, initial glenohumeral and subacromial arthroscopy is performed. Arthroscopic subacromial decompression is completed, and the cuff is inspected for a posterior cuff margin, and the presence of the long head of biceps. If amenable to reconstruction, an open approach to the shoulder is made. The presence of an irreparable rotator cuff tear is confirmed.

The infraspinatus tendon is elevated by sharp dissection from the posterior aspect of the greater tuberosity and tagged with a number of stay sutures. If amenable to separation in the intermuscular plane, the teres minor is left in situ provided sufficient infraspinatus tendon for reconstruction exists. The supraspinatus footprint is prepared with a high speed dental burr and rongeurs down to bleeding subcortical bone. The lateral aspect of the biceps sling is released, and the long head of biceps (LHB) is taken posterolaterally. A channel is prepared for the LHB using the burr and using 2 Super Revo anchors (Conmed Linvatec, Utica, NY) firmly tenodesed. The intraarticular portion of the LHB is left intact. 

Further 3 anchors are inserted in double row fashion at the footprint. The mobilized tendon is brought to the apex of the footprint, and Herculine (Conmed Linvatec, Utica NY) mattress sutures are placed from the superior LHB anchor through the central portion of the tendon. Further mattress sutures are placed from the anchors into the posterior half of the infraspinatus. With the arm in full adduction, the tendon is tensioned and firmly sutured down onto the footprint obtaining a double row repair. The leading edge and anterior half of the tendon is sutured onto the tenodesed LHB with interrupted Herculine sutures obtaining superior coverage of the humeral head. A small medial apical defect is usually apparent after the repair due to the angular position of the tendon transfer.

The approach to the shoulder is closed, with heavy sutures repairing the deltoid to the acromion in the case of a deltoid detaching approach. A Marcaine catheter pump is inserted into the subacromial space avoiding glenohumeral placement and subcutaneous tissues and skin are closed. A shoulder immobilizer is fitted to the patient.

## 3. Results

Between February 2005 and August 2007, 17 patients with ultrasound proven rotator cuff tears underwent shoulder arthroscopy and reconstruction. Age range was from 39 to 65 (mean 53). One patient had had a prior rotator cuff tear of the same shoulder, operatively treated 4 years previously. There were 6 Workers Compensation Board patients. Twelve patients were classed as manual or skilled manual workers, and the remaining 5 were employed in nonmanual occupations. Duration of symptoms prior to surgery ranged from 7 to 26 months.

Initial arthroscopy and subacromial decompression were performed in all patients. This confirmed the presence of a massive cuff tear. An open approach to the shoulder was made, initially via a modification of an anterior deltoid detaching approach (6 patients) and subsequently via a deltoid-splitting miniopen approach (11 patients).

In all cases, the rotator cuff was noted to have a massive tear, which was retracted, fixed, or significantly degenerate and was not amenable to primary repair. ITBIST reconstruction was performed, a deep marcaine catheter was left in place, and the wounds were closed. All patients underwent day-case surgery. 

Postoperative rehabilitation was performed according to a modification of the GOST protocol [1], with passive range of motion and isometric physiotherapy for weeks 0–6, active-assisted progressing to active range of motion for weeks 7–12, and resistance introduced at week 13.

Clinical followup was made at 6 weeks, 3, 4, 6, 12, and 20–24 months. Mean followup was 12 months (range 6 to 24 months). The Oxford Shoulder Score (OSS) was recorded preoperatively, at 3-4 months, 12 months and at final review. Mean preoperative OSS was 47.4 (range 35 to 58). Mean OSS at most recent review was 13.6 (range 12 to 23). In terms of pain relief, 11 (65%) patients were very satisfied with the final result, 5 (30%) patients were satisfied, 1 (5%) patient was unsatisfied. Average preoperative abduction was to 100 (range 30–170), and at last postoperative review this improved to 160 (range 90–180). According to the outcome measures of Bigliani et al. [2], there were 9 (54%) excellent, 7 (41%) good, and 1 (5%) fair results. At the time of last review, all patients had returned to work, with 15 returning to their prior level of occupation without modification. 

There were 2 complications. One patient developed a superficial wound infection which settled with oral antibiotics. One patient suffered a deltoid avulsion at 6 weeks postoperatively, and required surgical repair.

**Figure 1:**
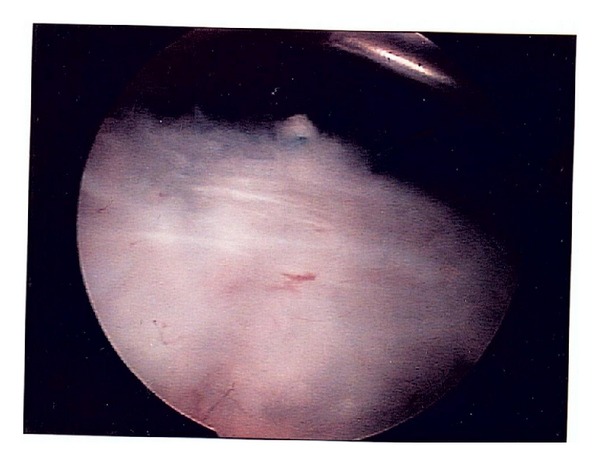
Intact tendon transfer at arthroscopic evaluation. Arthroscopic view from posterior portal showing intact transfer of infraspinatus onto long-head of biceps.

One patient had returned to work after full relief from symptoms, and fell onto the previously operated shoulder. Conservative management failed to improve his new symptoms, and he required repeat arthroscopy. This revealed a small partial thickness tear on the anterior border of the reconstruction, which was intact and showed excellent healing of the infraspinatus onto the long head of biceps (Figure 1). Surgical debridement of the partial tear resulted in rapid return to preinjury state.

## 4. Discussion

The management of rotator cuff tears has evolved significantly in the last 20 years. Tear size has been shown to correlate to outcome in terms of range, strength, and satisfaction [3]. In one series treated by standard repair, only 2 of 11 massive tears had excellent results, and only 6 of 11 much better [3]. Another series using modern open techniques reported a 15% unsatisfactory result [2]. A recent paper confirmed a 33% unsatisfactory outcome rate following partial repair of a massive cuff tear [4]. Many different techniques have been developed to address irreparable tears and improve final outcome. 

Advancement of the supraspinatus gave a 77% satisfactory result in a small series [5]. Mobilization of the supraspinatus and infraspinatus up to 3.5 centimetres has not been shown to injure the suprascapular nerve [6]. Medialisation of the supraspinatus more than 10 millimetres reduces the moment arm significantly and thus risks significant loss of power [7]. Margin convergence popularized by Burkhart has found favour, confirming that if biomechanical functionality of the cuff is reconstructed, despite full anatomic restoration not being achieved, then outcomes can be satisfactory.

The use of the biceps tendon has previously been documented in surgical reconstruction. Tenoplasty, by performing a widening of the biceps tendon and seating this onto the head to obtain coverage, was first described in 2001 [8]. Further studies confirmed the relief of pain obtained by this procedure [9], with 85.7% satisfactory results [8]. Performing a biceps tenodesis and utilizing the thickened free intraarticular portion to reconstruct a defect have been shown in two cases to provide satisfactory results [10]. Arthroscopically incorporating a biceps tenodesis into a rotator cuff repair has shown satisfactory results in 93% of 14 patients with biceps pathology coincident with rotator cuff tear [11]. Margin convergence to the biceps tendon, without tenodesis, was described by Burkhart, who proposed that this decreases strain on the partial repair [12]. 

Extrinsic tendon transfer has been widely investigated in the management of irreparable tears. Good results have been shown with this extensive procedure [13] and it is proposed that more strength is restored than with repair [14]. It is unclear whether latissimus dorsi or teres major transfers are better [13, 15, 16]. Subscapularis transfer has a number of potential concerns [17] and appears to be less suitable for posterosuperior cuff tears [16]. Deltoid flap reconstruction was reported to provide an 89% satisfaction rate [18], although this has not found favour in North America.

Infraspinatus transfer was shown in cadavers to be capable of covering a surgical supraspinatus defect with less tensile force than a subscapularis transfer [19]. Combined transfer of the teres minor and subscapularis was described with only 5 of 17 patients obtaining an excellent result, using a different outcome measure whereby excellent correlates to the good and excellent categories of R. J. Neviaser and T. J. Neviaser [20].

The technique described here appears to work well in the relief of symptoms and return to functionality following reconstruction of a massive cuff tear. It draws on the previously noted benefits of using the biceps tendon as part of the reconstruction; however, it differs in that the long head of biceps is used as a scaffold and vector couple, rather than simply providing soft-tissue interposition. It avoids the use of atrophic and degenerate retracted supraspinatus tissue, relying on more normal tendon for the reconstruction, and thus suggests better final functional outcome. It avoids the sizeable operation of an extrinsic latissimus or teres major transfer and is amenable to a miniopen deltoid splitting approach, thus preserving deltoid function and avoiding the complication of deltoid pull-off. Coverage of the humeral head is obtained; the reconstruction can function to depress the humeral head as well as acting as a posterior force couple. It is proposed that the tenodesed long head of biceps allows the vector pull of the transferred infraspinatus/teres minor to become more horizontal, producing a better humeral head depression force than the more oblique pull of a latissimus dorsi or teres major transfer.

These early results of a 95% of patients and clinical satisfactory outcome suggest that this is a useful operative procedure in the management of massive rotator cuff tears where other salvage procedures would otherwise have been considered.
